# Inhibition of glutamine uptake regulates mTORC1, glutamine metabolism and cell growth in prostate cancer

**DOI:** 10.1186/2049-3002-2-S1-P27

**Published:** 2014-05-28

**Authors:** Qian Wang, Rae-Anne Hardie, Seher Balaban, Mark Schreuder, Andrew Hoy, Michelle van Geldermalsen, Ladan Fazli, Rajini Nagarajah, Charles Bailey, John Rasko, Jeff Holst

**Affiliations:** 1 Origins of Cancer Laboratory, Centenary Institute, Camperdown, NSW, Australia; 2 Sydney Medical School, University of Sydney, NSW, Australia; 3 Discipline of Physiology, Bosch Institute, University of Sydney, NSW, Australia; 4 Boden Institute of Obesity, Nutrition, Exercise & Eating Disorders, University of Sydney, NSW, Australia; 5 Department of Urologic Sciences, University of British Columbia, Vancouver, BC, Canada; 6 Gene and Stem Cell Therapy Program, Centenary Institute, Camperdown, NSW, Australia; 7 Cell and Molecular Therapies, Royal Prince Alfred Hospital, Camperdown, NSW, Australia

## Background

Amino acids such as glutamine are important for tumor cell growth, survival and metabolism. There is renewed interest in glutamine metabolism due to the importance of reductive carboxylation in cancer. The amino acid transporter ASCT2 (SLC1A5) mediates uptake of glutamine in cancer cells. We have recently reported that ASCT2 expression is significantly upregulated in melanoma, and ASCT2 inhibition significantly decreases glutamine uptake, cell growth, cell cycle and mTORC1 pathway activation [1]. We have previously shown that ASCT2 expression is regulated by the androgen receptor in prostate cancer [2], and in this current study we further examine ASCT2 expression levels in prostate cancer. Our specific aim was to determine the impact of inhibiting ASCT2-mediated glutamine uptake and metabolism on cell growth.

## Materials and methods

We have assessed the role of ASCT2 in prostate cancer using: (1) tissue microarray analysis of ASCT2 protein expression in patients before and after neoadjuvant hormone therapy, (2) cell lines (LNCaP and PC-3) and (3) xenograft (PC-3) models *in vivo*. Glutamine uptake, cell growth, cell cycle, mTORC1 pathway and glutamine metabolism pathways were assessed using a variety of ASCT2 inhibitors and shRNA mediated ASCT2 knockdown.

## Results

ASCT2 is highly expressed in primary prostate cancer, but levels decrease after neoadjuvant hormone therapy, before increasing again in recurrent disease. Inhibition of ASCT2 function by benzylserine led to decreases in glutamine uptake, glutamine metabolism (oxygen consumption rate, glutamine oxidation and lipogenesis), cell cycle progression, mTORC1 pathway activation and cell growth. These data were confirmed after shRNA-mediated ASCT2 knockdown *in vitro*. Furthermore, shRNA knockdown in PC-3 cell xenografts led to a significant reduction in tumor growth *in vivo*.

## Conclusions

ASCT2-mediated glutamine uptake is essential for multiple pathways including glutamine metabolism and mTORC1 signaling, thereby regulating cellular energy, protein synthesis and cell growth. As such, ASCT2 is a putative therapeutic target in prostate cancer.
